# The next frontier in osseointegration: energy and speed as critical determinants and their enhancement by UV photofunctionalization

**DOI:** 10.1186/s40729-025-00638-2

**Published:** 2025-08-04

**Authors:** Takanori Matsuura, Keiji Komatsu, Rune Shibata, Toshikatsu Suzumura, Justin Choi, Takahiro Ogawa

**Affiliations:** 1 Weintraub Center for Reconstructive Biotechnology, UCLA School of Dentistry, Los Angeles, CA US; 2 Department of Periodontology, Graduate School of Medical and Dental Sciences, Institute of Science Tokyo (Science Tokyo), Bunkyo-ku, Tokyo, Japan; 3 Department of Lifetime Oral Health Care Sciences, Graduate School of Medical and Dental Sciences, Institute of Science Tokyo (Science Tokyo), Bunkyo- ku, Tokyo, Japan; 4 Division of Regenerative and Reconstructive Sciences, UCLA School of Dentistry, Los Angeles, CA US

**Keywords:** Titanium implant, Osseointegration, Biomechanical test, Ultraviolet light, Photofunctionalization

## Abstract

**Purpose:**

This study aimed to redefine biomechanical understanding of osseointegration by dissecting its multi-faceted nature—strength, stiffness, energy, and the speed of development—through in vivo analysis

**Methods:**

Titanium implants were placed bilaterally in rat femurs, with one side receiving ultraviolet (UV) photofunctionalization and the contralateral side serving as untreated control. Biomechanical push-in tests were performed at day 4, and weeks 1 to 3 post-implantation. Yield strength, elastic modulus, toughness, and energy were measured over time, and their rates of change were calculated to assess dynamic progression. Mineralization at the bone–implant interface was quantified using energy-dispersive X-ray spectroscopy.

**Results:**

Yield strength followed a second-degree growth curve, plateauing over time in control implants. Derivative analysis revealed that the speed of strength gain peaked early and then declined. In contrast, osseointegration energy steadily increased throughout healing, with its rate of gain accelerating over time. Yield strength correlated quadratically with peri-implant mineralized tissue, indicating early saturation, while energy and toughness showed a continuous linear relationship. UV photofunctionalization enhanced all biomechanical parameters. Notably, it accelerated early strength acquisition (up to 3-fold) and markedly increased both energy (up to 3.4-fold) and its rate of development (up to 4.9-fold).

**Conclusions:**

By introducing energy and its speed as novel indices, this study offers a more dynamic and functionally relevant framework for evaluating osseointegration. UV photofunctionalization not only accelerates early mechanical stability but amplifies energy acquisition at the bone–implant interface, promoting faster and more robust development of mechanical resilience.

**Graphical Abstract:**

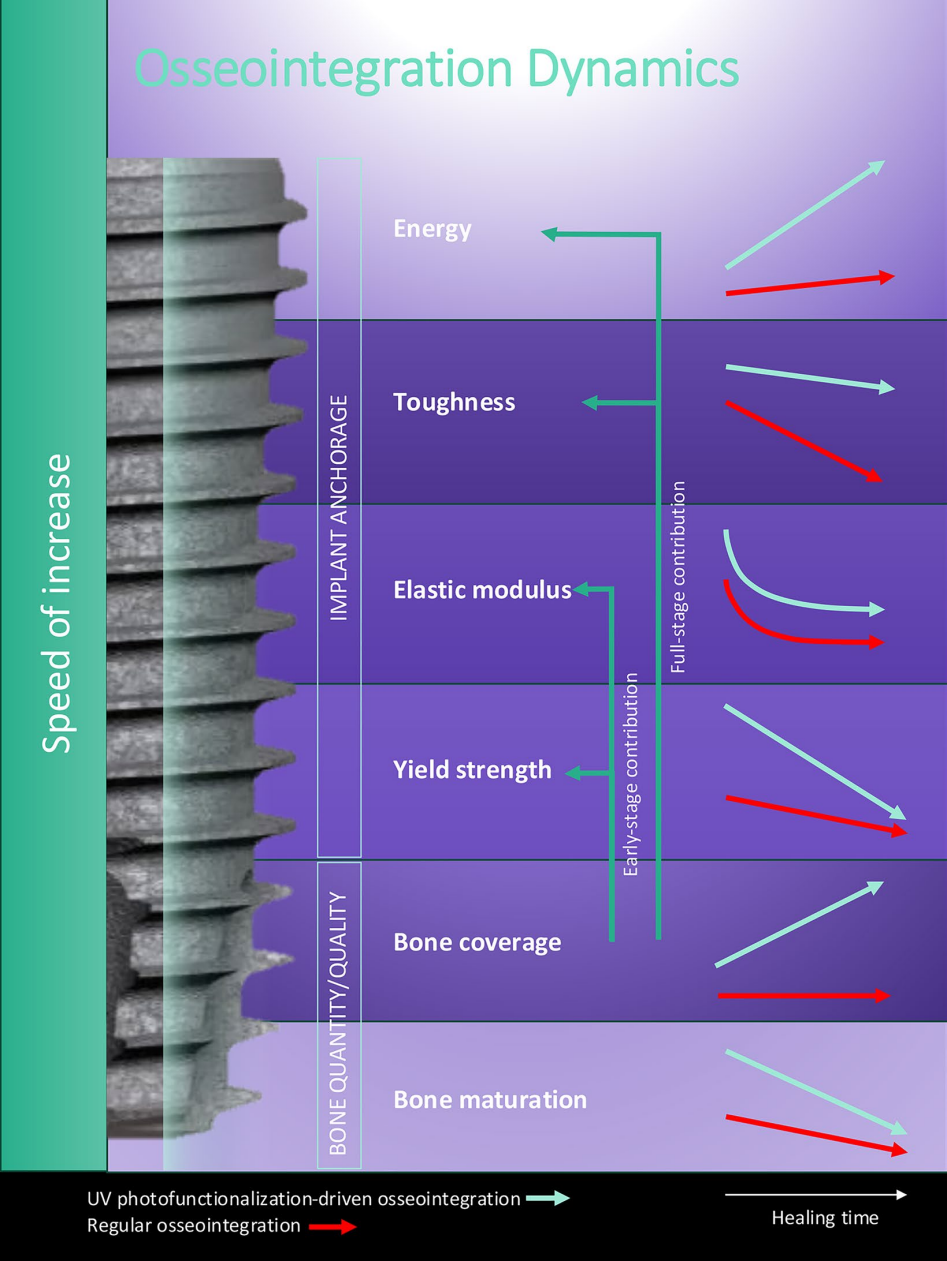

##  Background

The long-term success of titanium implants—whether in orthopedic or dental applications—hinges on their capacity to achieve robust, stable anchorage within bone [1–5]. This process, known as osseointegration, entails a complex interplay between bone bonding and de novo bone formation around the implant surface [6–9]. Although implant therapy has become a cornerstone of modern clinical practice, our understanding of the biomechanical underpinnings of osseointegration remains fundamentally incomplete [9–13]. Notably, progress in surface design and integration-enhancing technologies has plateaued, due in part to a narrow focus on static measures of success and a failure to address the temporal and mechanical complexity of the integration process [7, 13–15].

A critical, overlooked dimension in implant research is the dynamic progression and mechanical quality of osseointegration—how it evolves over time, and how its various facets contribute to long-term functionality and stability. Indeed, biomechanical quality of bone and its adhesive ability to titanium can be modulated by surface topography of implants [16–18]. Conventional studies primarily assess yield strength, a singular mechanical indicator defined as the load at which the bone–implant interface begins to fail [19–23]. While important, this measure captures only a fraction of what implants experience in vivo. Clinical scenarios are dominated not by isolated peak loads, but by continuous, repetitive forces—such as chewing cycles in the oral cavity or gait-related loading in orthopedic applications. Regulatory fatigue testing requirements reflect this: dental implants, for example, must endure 200,000 cycles at just ~ 200 N, far below actual occlusal peak forces and breakpoint load of the implant itself. These discrepancies emphasize a critical gap in current evaluation standards: strength alone does not equate to durability.

To address this, we propose a shift in focus toward energy absorption, or osseointegration energy—a holistic mechanical parameter that reflects both the resistance to force and the capacity to deform without failing. This metric captures the interface’s ability to manage and dissipate repetitive mechanical stress, offering a more clinically relevant indicator of durability. Despite its clear importance, energy absorption has been virtually absent from osseointegration studies. Existing biomechanical assays—push-in [24–29], pull-out [30], tensile [17, 18, 31–33], removal torque [19, 20], shear [22]—primarily report strength, ignoring the capacity of the bone–implant complex to sustain function under cyclic loading. This omission leaves a fundamental question unanswered: not just how strong is osseointegration, but how resilient is it?

Equally critical is the concept of speed—how rapidly osseointegration develops. Most studies rely on static, cross-sectional measurements taken at fixed time points, failing to capture the rate at which mechanical properties emerge and mature [34–38]. Yet, the speed of strength, stiffness, and energy gain is a decisive factor in determining when implants can be safely loaded and how long-term outcomes unfold. Without understanding these kinetics, clinicians operate on empirical or overly conservative protocols. A dynamic, rate-based approach would provide a rational, evidence-based foundation for optimizing early loading strategies and surface engineering efforts.

The surface properties of titanium implants play a decisive role in osseointegration [14, 39–41]. Various methods, including surface roughening/texturing [42–47] and chemical [48–50] or physicochemical modifications [51–53], have been explored to enhance implant performance. Among physicochemical modifications, UV photofunctionalization has emerged as a powerful tool [54–59]. By removing organic contaminants and restoring surface hydrophilicity, short-duration UV treatment transforms titanium surfaces into highly osteoconductive platforms [60–64]. UV-treated implants attract osteoblasts, enhance cell attachment and proliferation, and significantly increase early-stage yield strength of implants in vivo [63–67]. However, its broader impact—particularly on the durability, resilience, and temporal dynamics of osseointegration—remains uncharted.

This study presents a comprehensive, time-resolved biomechanical framework for assessing osseointegration. Moving beyond conventional yield strength measurements, we evaluate additional functional parameters including elastic modulus (stiffness), toughness (resistance to failure), energy absorption (mechanical resilience), and—critically—the rate at which each of these properties develops over time. Using a rat femur model and serial biomechanical push-in testing, we capture the dynamic progression of these metrics from early healing to maturation. We also examine the influence of peri-implant mineralization and demonstrate how UV photofunctionalization fundamentally reshapes the trajectory of osseointegration. Through this expanded and temporally focused approach, the study redefines the biomechanical understanding of implant integration.

## Methods

### Implant Preparation and surface characterization

**Fig. 1:**
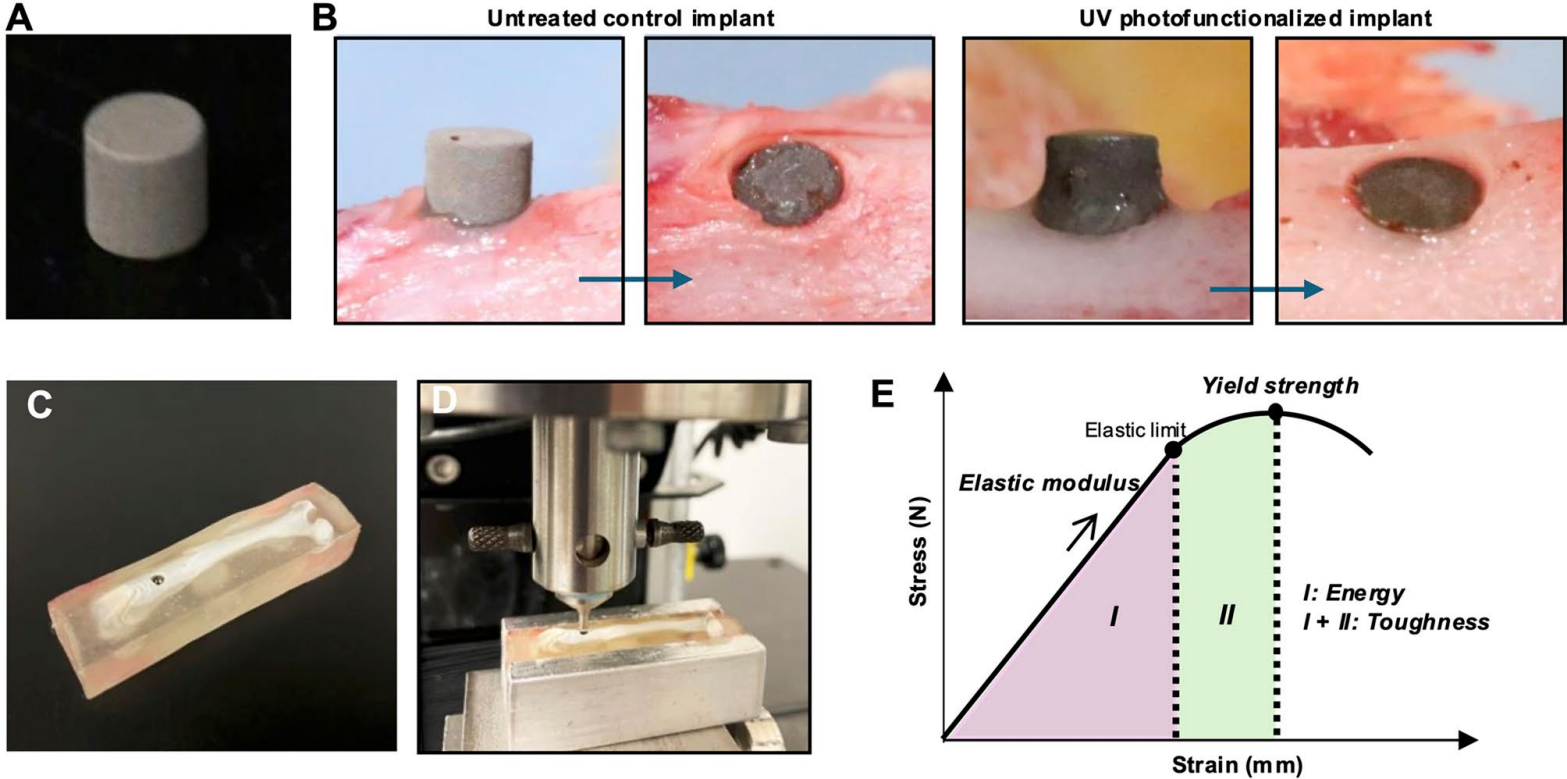
Materials and experimental protocols used in this study. (A) Cylindrical titanium implants utilized for biomechanical testing. (B) Surgical placement of implants into the rat femur. Hemophobic control implants were converted to hemophilic surfaces through 1-minute UV photofunctionalization prior to implantation. (C) Retrieved femur after designated healing period, embedded in an acrylic resin block with the implant surface oriented parallel to the ground. (D) Mounted femur block positioned in a mechanical testing apparatus for the implant push-in test. (E) Schematic representation of the stress-strain curve generated during the push-in test, illustrating the definitions of biomechanical parameters evaluated in this study

Cylindrical implants (2 mm diameter × 2 mm length) were machined from medical-grade commercially pure titanium (Grade 4). All implants underwent standard sandblasting and acid-etching procedures to produce microrough surface topography (Fig. 1A). UV photofunctionalization was performed using vacuum UV (VUV) light at a wavelength of 172 nm [68–73] for one minute (UV Activator, DIO IMPLANT, Busan, Korea). Surface morphology was analyzed using scanning electron microscopy (SEM; Supra 40VP, Zeiss, Oberkochen, Germany). Surface elemental composition was evaluated using X-ray photoelectron spectroscopy (XPS). Wettability was assessed by measuring the contact angle of 0.5 µL droplets of deionized water (ddH₂O) and rat whole blood on each surface.

### Surgical protocol for implant placement

The UCLA Institutional Biosafety Committee (BUA-2-22-036-001) approved the study protocol. Twenty-four male Sprague–Dawley rats, aged twelve-week, were used for in vivo implantation. Analgesia was administered 60 min prior to surgery (Buprenorphine Hydrochloride, Par Pharmaceutical, Chestnut Ridge, NY), and anesthesia was induced and maintained with 1–2% isoflurane inhalation. A full-thickness skin and muscle incision was made to expose the distal femur. Implant sockets were prepared 10 mm from the distal end using a 0.8 mm round bur followed by a 2 mm pilot drill (Fig. 1B). Implants were placed passively at the bone level.

Using a split-leg design, each rat received a control (non-UV-treated) implant in one femur and a UV-photofunctionalized implant in the contralateral femur, with the side of treatment alternated among animals. Muscles were closed with absorbable sutures (5 − 0 polyglycolic acid, McKESSON, Irving, TX), and skin closure was achieved with non-resorbable monofilament sutures (4 − 0 nylon, McKESSON). Post-operative analgesia was administered subcutaneously 6 h after surgery and again the following morning.

### Biomechanical push-in test and analytical metrics

Implant anchorage was evaluated by biomechanical push-in testing [24–26] at day 4, and weeks 1, 2, and 3 post-implantation. At each time point, six rats were euthanized via CO₂ asphyxiation, and femurs containing implants were harvested and embedded in auto-polymerizing acrylic resin (Teets Cold Cure Denture Material, Co-Oral-Ite Dental Mfg Co., Diamond Springs, CA), ensuring the implant top surface was oriented horizontally (Fig. 1C). A universal testing machine (Instron 5544, Instron, Canton, MA) equipped with a 2000 N load cell and a 1.9 mm diameter stainless steel rod applied vertical downward force to the implant at a crosshead speed of 1 mm/min (Fig. 1D). Force-displacement data were recorded and converted to stress–strain curves (Fig. 1E). The following biomechanical parameters were extracted:

Yield strength: The maximum load recorded (peak of the stress–strain curve).

Elastic modulus: The slope of the linear region of the curve up to the elastic limit.

Energy: The area under the curve up to the elastic limit, representing the capacity of the interface to absorb mechanical energy prior to plastic deformation.

Toughness: The area under the curve up to the yield point, representing resistance to failure initiation at the interface.

It is important to note that these definitions were adapted to the biological context of the bone–implant interface. In this push-in model, a definitive fracture point at implant–tissue interface could not be reliably detected; thus, the yield point was used as a practical threshold to define toughness. Similarly, energy was defined as the elastic energy absorbed prior to yielding to the plastic phase.

Each parameter was plotted over time, and best-fit curves were derived via regression analysis. To evaluate the dynamics of osseointegration, derivative analysis was performed on each regression equation to calculate the rate of change—termed “speed” metrics. These speed values, while derived from the same datasets as the original measurements, offer a complementary perspective by quantifying the temporal development of each biomechanical property.

### Implant interface morphological and elemental analyses

The surface morphology and elemental composition of retrieved implants were analyzed using SEM and energy-dispersive X-ray spectroscopy (EDX; Noran System 6, Thermo Fisher Scientific). The calcium-to-titanium (Ca/Ti) ratio was calculated to assess the extent of mineralized tissue formation at the implant interface, while the calcium-to-phosphorus (Ca/P) ratio was used as an indicator of mineralized tissue maturation. Correlation analyses were conducted between each biomechanical parameter and the Ca/Ti ratio.

### Statistical analysis

For the biomechanical push-in test, six implants were used per group (untreated control and UV-photofunctionalized; *n* = 6). For wettability, XPS, and interfacial morphological and elemental analyses, three implants per group were evaluated (*n* = 3). Statistical analyses were conducted using GraphPad Prism (version 10.2.3; GraphPad Software, Boston, MA). Comparisons between groups were made using paired t-tests. A p-value of < 0.05 was considered statistically significant.

## Results

### *Surface properties of titanium implants*.

**Fig. 2:**
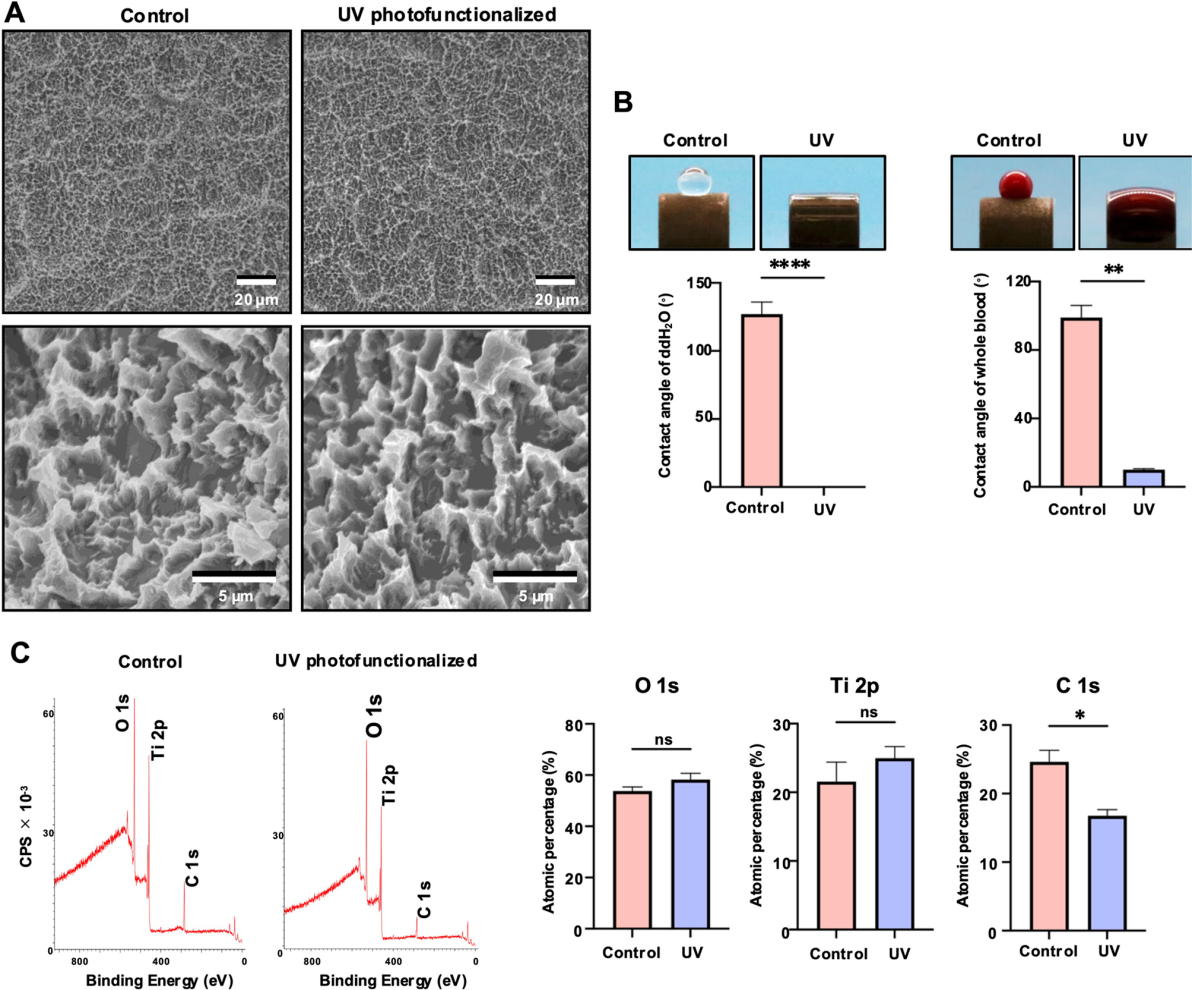
Surface characterization of titanium implants. (A) Low- and high-magnification SEM images of untreated and UV-photofunctionalized implants. UV photofunctionalization was performed using VUV light for one minute. (B) Hydrophobic/hydrophilic and hemophobic/hemophilic properties of the implant surfaces. Side-view images show 0.5 µL of ddH₂O and rat whole blood placed on the implant surfaces, along with their respective contact angles. (C) Chemical composition analysis by X-ray photoelectron spectroscopy (XPS), detecting titanium (Ti), oxygen (O), and carbon (C) on the implant surfaces. *p < 0.05, **p < 0.01, ****p < 0.0001; statistically significant differences between control and UV-photofunctionalized implants

The titanium implants used in this study exhibited microrough surface features characteristic of acid-etched titanium, consisting of 1–5 μm pits or compartmental architectures (Fig. 2A). No morphological differences were observed between the control and UV photofunctionalized titanium surfaces. While the control implants were hydrophobic and hemophobic, with their contact angle greater than 90°, UV photofunctionalized implants were hydrophilic and hemophilic, with a contact angle less than 10° (Fig. 2B). The atomic percentage of carbon, initially 25% on untreated control implants, was significantly reduced to 15% after UV photofunctionalization (Fig. 2C).

### *Yield strength of osseointegration and its speed of increase*.

**Fig. 3:**
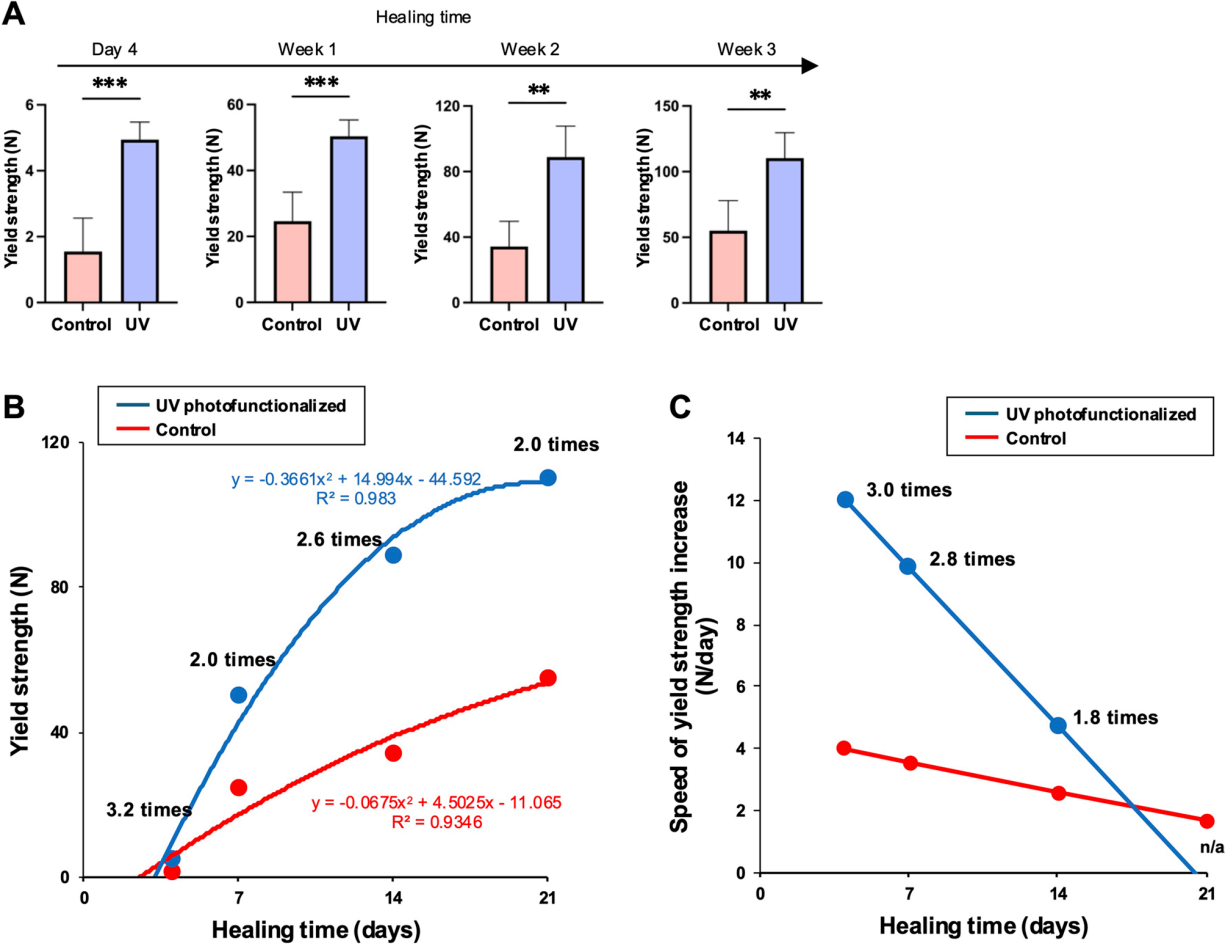
Yield strength of osseointegration for control and UV-photofunctionalized implants. Implants placed in rat femurs were evaluated at 4 days, 1 week, 2 weeks, and 3 weeks post-implantation using a biomechanical push-in test. (A) Yield strength, defined as the maximum point in the stress-strain curves. See Fig. 1 for definition. **p < 0.01, ***p < 0.001; statistically significant differences between control and UV-photofunctionalized implants. (B) Temporal progression of yield strength, with best-fit curves obtained through second-degree regression analysis for both groups. Regression equations are included. (C) Speed of yield strength increase, calculated through derivative analysis of the regression curves shown in panel B. This analysis quantifies the rate of mechanical strength acquisition over time. Note: Panels A–C present complementary analytical perspectives based on the same experimental dataset, with derivative analysis serving to extract temporal dynamics from the primary measurements

The biomechanical properties of osseointegration were assessed using an implant push-in test in a rat femur model. Yield strength was measured from the stress-strain curve. Both control and UV photofunctionalized implants exhibited an increase in yield strength with healing time, with a significant difference between the two groups, ranging from 2.0 times to 3.2 times (Fig. 3A and B). Regression analysis fit a second-degree curve to the temporal change in yield strength for both groups (Fig. 3B), representing a rapid increase for UV photofunctionalized implants during the early (week 1) to mid (week 2) healing stages, followed by a plateau in both groups during the late healing stage (week 3).

To assess the dynamics of yield strength development, derivative analysis was performed on the regression curves, producing first-degree equations that represent the rate—or speed—of yield strength gain over time (Fig. 3C). The speed of increase peaked at the initial stage (day 4) and declined linearly as healing progressed. UV photofunctionalization significantly enhanced this early-phase development, with the speed of yield strength increase being 3.0-fold, 2.8-fold, and 1.8-fold higher than controls at the initial (day 4), early (week 1), and mid (week 2) stages, respectively.

#### **Stiffness of osseointegration and its speed of development**.

**Fig. 4:**
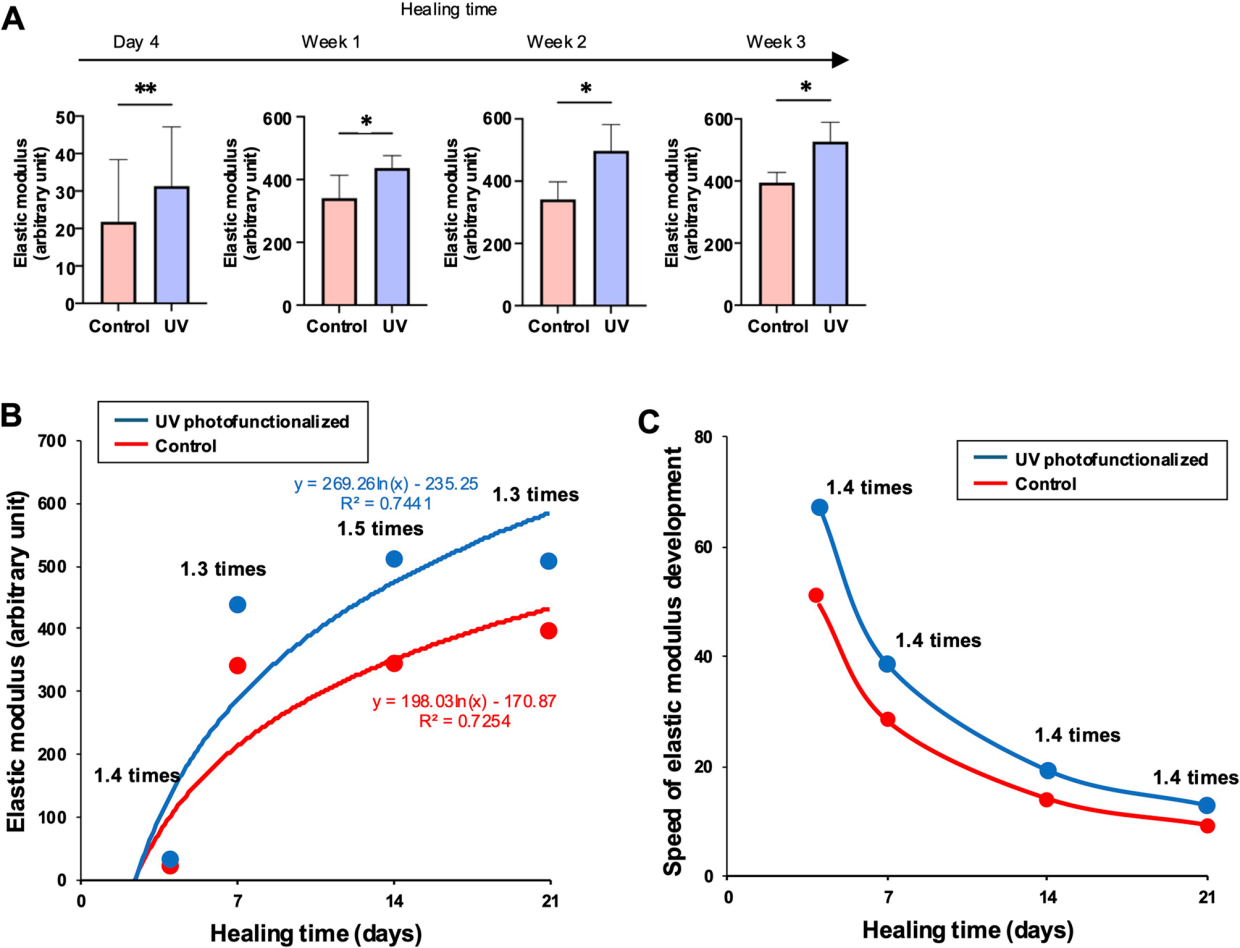
Elastic modulus of osseointegration, reflecting the stiffness of the bone-implant interface. (A) Elastic modulus, determined as the slope of the stress-strain curve within the elastic deformation region. Refer to Fig. 1 for definition. *p < 0.05, **p < 0.01; statistically significant differences between control and UV-photofunctionalized implants. (B) Elastic modulus as a function of healing time, with regression curves illustrating the trend for each group. The corresponding regression equations are included. (C) Speed of elastic modulus development, calculated through derivative analysis of the regression curves shown in panel B. Note: Panels A–C present complementary analytical perspectives based on the same experimental dataset, with derivative analysis serving to extract temporal dynamics from the primary measurements

The elastic modulus was consistently higher for UV photofunctionalized implants throughout the healing process (Fig. 4A). The temporal dynamics of stiffness followed a logarithmic curve for both implant types, showing a rapid increase up to the early healing stage, followed by saturation (Fig. 4B). The speed of development in elastic modulus, obtained by derivative analysis, was inversely proportional to healing time for both implants (Fig. 4C). The speed dropped rapidly from the initial to early stages and stabilized during the mid-to-late stages. The elastic modulus increase speed remained consistently higher for UV photofunctionalized implants.

#### **Energy of osseointegration and its speed of accumulation**.

**Fig. 5:**
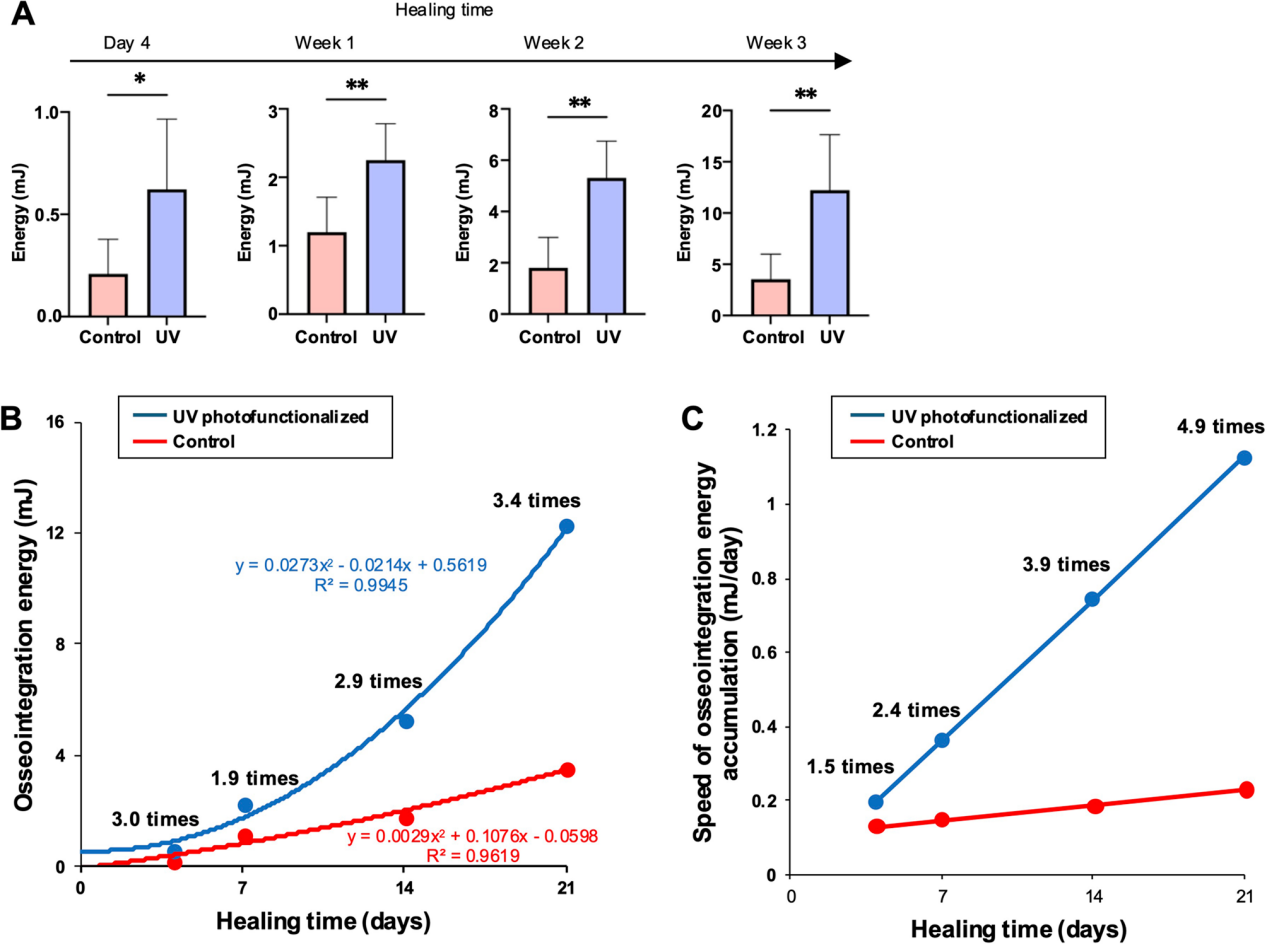
Osseointegration energy, representing the ability of the bone-implant interface to absorb mechanical load and displacement before transitioning into plastic deformation. (A) Osseointegration energy, quantified as the area enclosed within the elastic zone of the stress-strain curve or obtained through integral calculations applied to this region. See Fig. 1 for definition. *p < 0.05, **p < 0.01; statistically significant differences between control and UV-photofunctionalized implants. (B) Time-dependent changes in osseointegration energy, illustrated with regression curves for each implant surface. Regression equations are also provided. (C) Speed of osseointegration energy accumulation, quantified by derivative analysis of the regression curves in panel B. This metric describes the rate at which the interface gains mechanical energy-absorbing capacity over time. Note: Panels A–C provide complementary analytical views derived from the same experimental dataset. Derivative analysis in panel C offers insight into the dynamic evolution of energy development beyond the absolute values shown in panels A and B

Energy absorption capacity reflects the ability of the bone–implant interface to withstand mechanical forces before undergoing permanent deformation. A higher energy absorption denotes a more resilient and durable integration under prolonged mechanical stress. Unlike yield strength and elastic modulus, the energy of osseointegration followed a distinctly different dynamic trajectory. Modeled by second-degree curves, osseointegration energy increased progressively with healing time, displaying a more pronounced and sustained rise during the later stages of healing in both implant groups (Fig. 5A and B). UV-photofunctionalized implants consistently exhibited higher energy values, with differences reaching up to 3.4-fold compared to control implants by week 3.

To analyze the temporal dynamics of energy development, derivative analysis was applied to the regression curves (Fig. 5C). This revealed a clear contrast to the decelerating trends observed in yield strength and elastic modulus. In both groups, the speed of energy accumulation increased continuously throughout the healing period. UV photofunctionalization significantly amplified this rate of development, producing a steeper first-degree curve and a more rapid trajectory. The speed of energy accumulation in UV-treated implants reached up to 4.9 times that of controls, indicating not only a greater overall energy capacity but also a fundamentally accelerated maturation process. These findings suggest that UV photofunctionalization enhances both the magnitude and momentum of energy-based integration, contributing to a more robust and functionally resilient implant interface.

#### **Toughness of osseointegration and its speed of increase**.

**Fig. 6:**
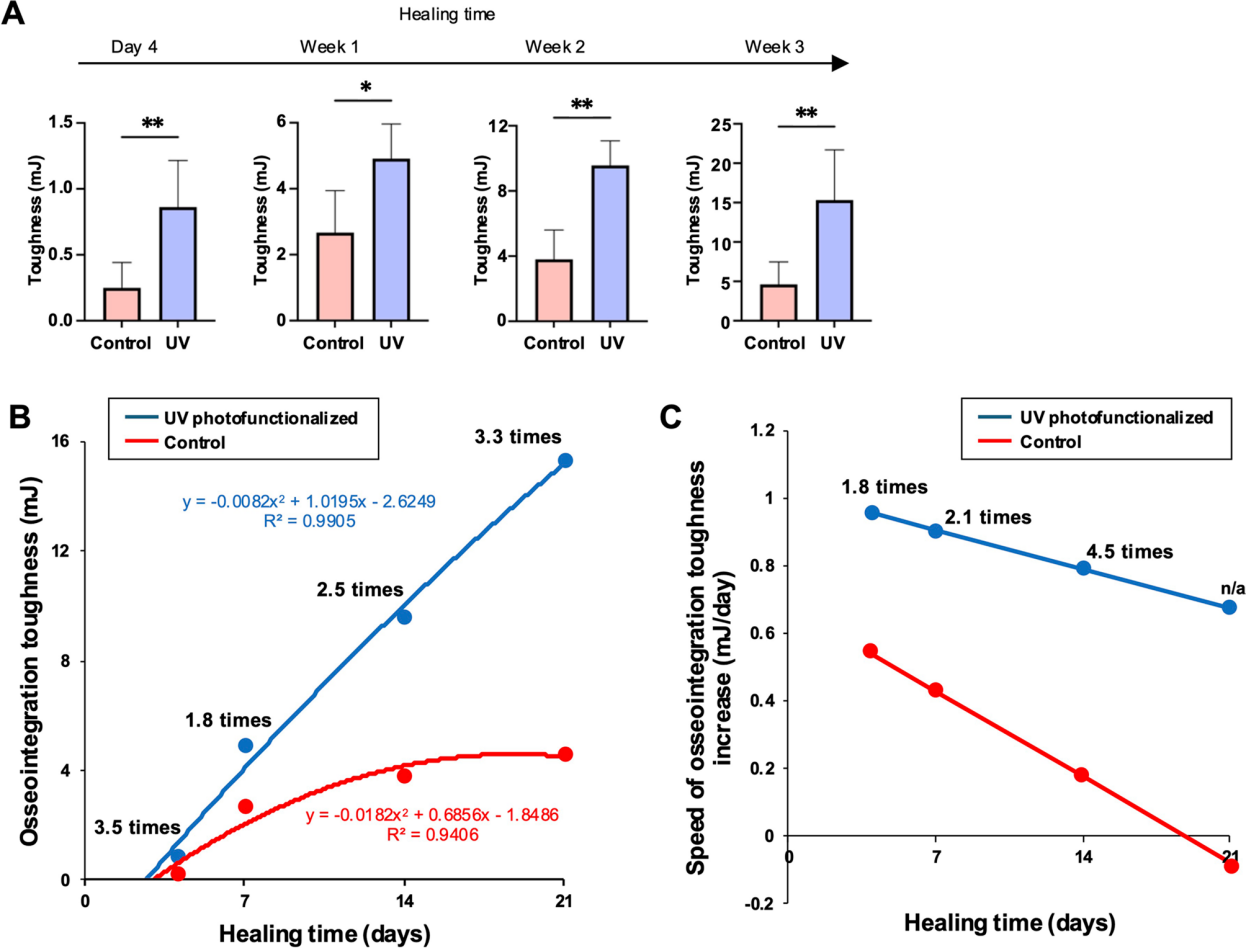
Osseointegration toughness, indicating the total mechanical capacity of the bone-implant interface to endure load and displacement before interfacial failure. (A) Osseointegration toughness measured as the area under the stress-strain curve up to the rupture point, incorporating both elastic and plastic deformation zones. See Fig. 1 for definition. *p < 0.05, **p < 0.01; statistically significant differences between control and UV-photofunctionalized implants. (B) Osseointegration toughness progression over time, displayed with regression-based trend lines for untreated and UV-photofunctionalized implants. Corresponding regression equations are provided. (C) Speed of toughness increase, calculated via derivative analysis of the regression curves in panel B, capturing the rate at which interfacial fracture resistance evolves over time. Note: Panels A–C offer complementary analytical perspectives derived from the same experimental dataset. Derivative analysis in panel C reveals dynamic trends in toughness development not apparent from absolute values alone

Toughness, or fracture resistance, represents the total force the bone–implant interface can withstand before complete structural failure. A high yield strength without toughness means the interface may be strong but brittle and prone to catastrophic failure. The osseointegration toughness displayed a dynamic trajectory of second-degree equations closely resembling that of energy absorption. However, a key divergence was observed: in control implants, the toughness curve plateaued during the mid-to-late healing stages, whereas UV-photofunctionalized implants exhibited a continuous upward trend throughout the healing period (Fig. 6A and B).

This sustained increase resulted in a progressively widening gap in toughness between the two groups, with UV-treated implants demonstrating increasingly superior performance as healing advanced. Although the rate of toughness increase declined over time for both groups, the decrease was substantially less pronounced in UV-photofunctionalized implants (Fig. 6C). These findings suggest that UV treatment not only elevates the peak toughness of osseointegration but also maintains its development over an extended healing window—supporting more durable and failure-resistant bone–implant integration over time.

#### **Morphology of the implant-tissue interface**.

**Fig. 7:**
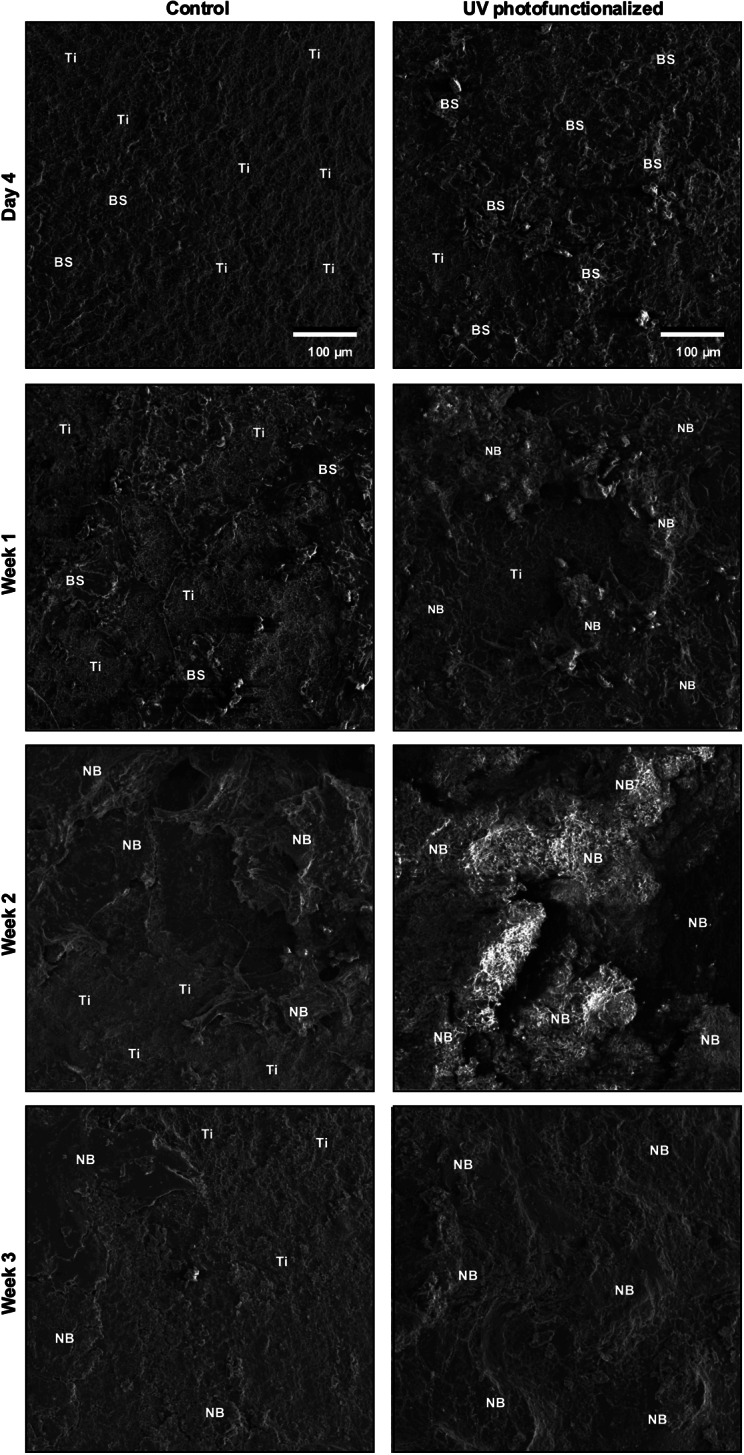
Tissue morphology at the implant interface during healing. SEM images of retrieved implants at 4 days, and 1, 2, and 3 weeks post-implantation. Ti: original titanium implant surface; BS: biological structure; NB: newly formed bone-like structure

Implants retrieved at each healing time point were examined for their interfacial morphology and tissue composition. On day 4, control implants exhibited largely exposed microrough titanium surfaces with minimal biological coverage (BS in Fig. 7). In contrast, UV-photofunctionalized implants showed substantial surface coverage by biological structures, indicating earlier initiation of tissue interaction. By week 1, three-dimensional deposition of bone-like tissue (NB in Fig. 7) was evident only on UV-photofunctionalized implants. Control implants displayed limited areas of biological coverage, with tissue lacking defined morphology and extensive regions of exposed titanium still visible.

At week 2, bone-like tissue formation became more pronounced in both groups; however, UV-photofunctionalized implants presented a denser and more developed trabecular-like architecture. While control implants retained noticeable areas of uncovered surface, UV-treated implants were nearly fully enveloped by newly formed tissue. By week 3, UV-photofunctionalized implants demonstrated almost complete coverage with bone-like tissue, signifying advanced integration. In contrast, control implants continued to exhibit incomplete coverage and visible titanium. Notably, the bone-like tissue at this stage appeared thinner and more layered compared to the robust trabecular structures observed at week 2, suggesting a transition from initial reactive bone formation toward lamellar bone remodeling.

#### **Chemistry of the implant-tissue interface**.

**Fig. 8:**
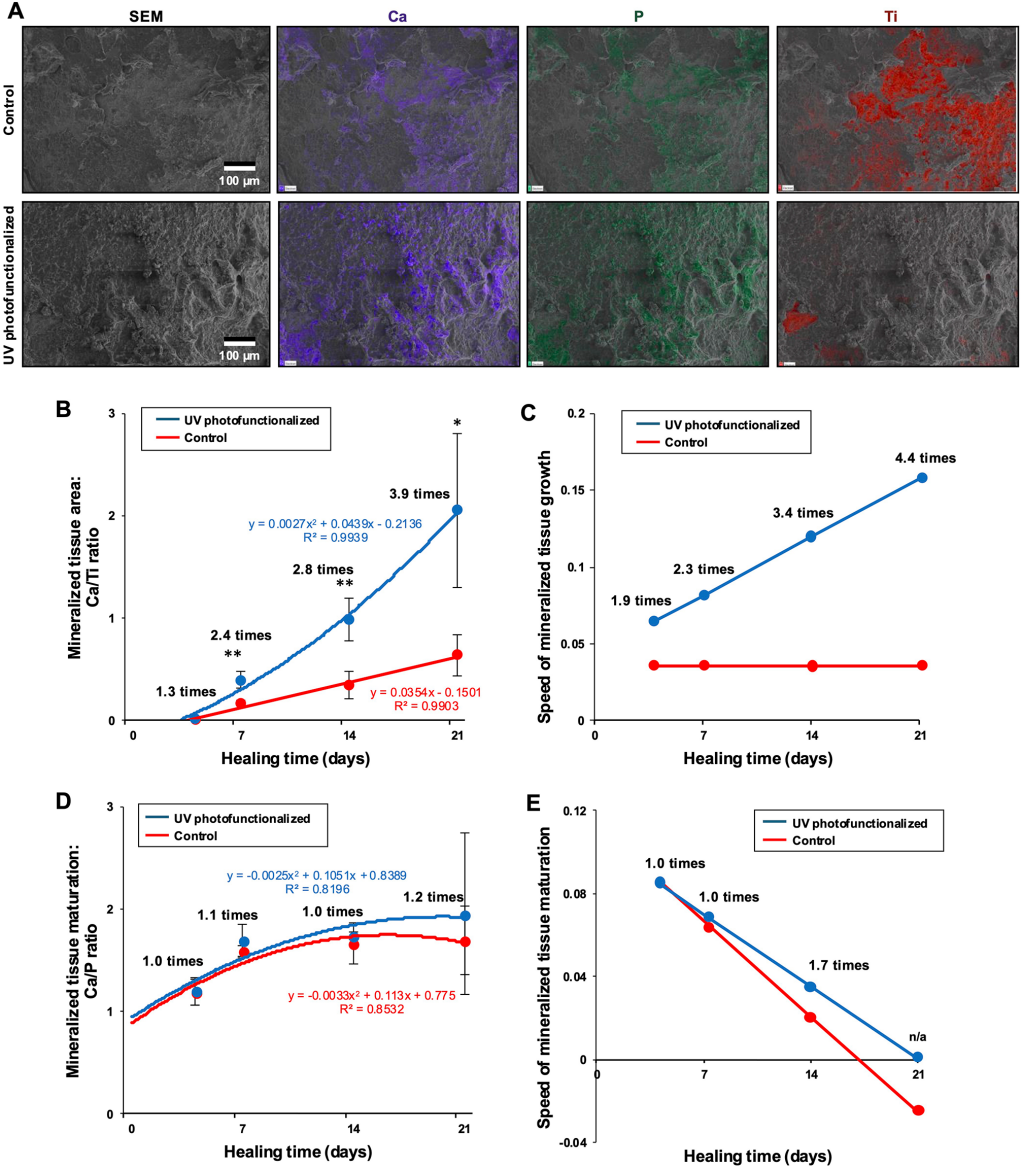
Mineralization characteristics at the implant interface. (A) SEM images of the implant interface at 2 weeks of healing, shown alongside superimposed EDX elemental maps for titanium, calcium, and phosphate. (B) Mineralized tissue area on implant surfaces, quantified as the Ca/Ti ratio via EDX analysis, plotted over healing time. Regression curves and equations fitting the data are included. *p < 0.05, **p < 0.01; statistically significant differences between control and UV-photofunctionalized implants. (C) Speed of mineralized tissue growth, determined by derivative analysis of the regression equations in panel B. (D) Mineralized tissue maturation, assessed using the Ca/P ratio, plotted against healing time. Corresponding regression curves and equations are provided. (E) Speed of mineralized tissue maturation, calculated by derivative analysis of the regression equations in panel D

Energy-dispersive X-ray spectroscopy (EDX) revealed the presence of calcium (Ca) and phosphorus (P) in the interfacial tissue of both implant groups, as demonstrated in the representative elemental overlay maps from week 2 (Fig. 8A). Ca and P signals were markedly stronger and more widespread on UV-photofunctionalized implants, indicating more extensive mineralized tissue formation. Correspondingly, titanium (Ti) signals were significantly attenuated, reflecting reduced surface exposure due to advanced tissue coverage.

The extent of mineralization was quantified using the Ca/Ti ratio (Fig. 8B). On untreated control implants, mineralized tissue formation increased linearly over time, following a first-degree equation. In contrast, UV-photofunctionalized implants exhibited exponential growth in mineralized coverage, fitting a second-degree equation. As a result, the disparity in mineralized tissue area between the two groups progressively widened with time: UV-treated implants showed 1.3 times, 2.4 times, 2.8 times, and 3.9 times greater mineralized tissue coverage at day 4, week 1, week 2, and week 3, respectively.

Derivative analysis of these equations further highlighted the distinct growth dynamics between the groups (Fig. 8C). While the mineralization rate remained constant for control implants throughout healing, UV-photofunctionalized implants demonstrated an accelerating growth rate, reaching a 4.4 times higher mineralization speed by week 3.

Tissue maturation was assessed via the Ca/P ratio derived from EDX (Fig. 8D). Both groups exhibited a second-degree increase in Ca/P ratio over time, gradually approaching a plateau. Although the final Ca/P values were comparable, differentiation of the growth curves (Fig. 8E) revealed that UV-photofunctionalized implants consistently achieved a higher rate of mineral maturation during the healing process.

### *Disproportionate contributions of mineralized tissue formation to osseointegration metrics*.

The divergent temporal patterns observed in yield strength, elastic modulus, energy, and toughness prompted an investigation into how each biomechanical parameter correlates with mineralized tissue formation—recognized as a central determinant of osseointegration.

**Fig. 9:**
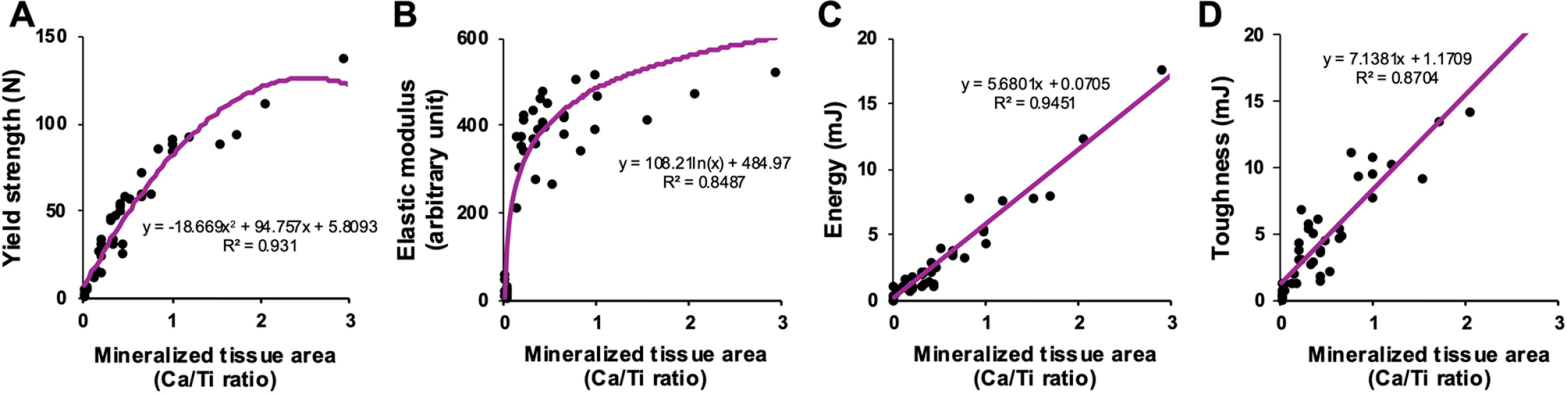
Divergent trajectories of osseointegration metrics and their dependence on mineralized tissue formation. Yield strength (A), elastic modulus (B), energy (C), and toughness (D) are plotted against mineralized tissue area (Ca/Ti ratio), illustrating their dependence on bone formation at the interface. Regression curves and equations are included

Regression analysis revealed that yield strength displayed a second-degree correlation with mineralized tissue area (expressed by the Ca/Ti ratio), while elastic modulus followed a logarithmic trend (Fig. 9). Both parameters showed steep increases during the early stages of mineral deposition, but plateaued as mineralized tissue continued to accumulate. This saturation effect suggests that once a critical level of mineralization is reached, additional tissue coverage contributes minimally to further mechanical strengthening or stiffness.

In contrast, osseointegration energy and toughness demonstrated consistent linear correlations with mineralized tissue area. These metrics followed first-degree equations, indicating a direct and proportional relationship between the extent of mineralization and the interface’s ability to absorb mechanical forces and resist fracture. Unlike yield strength and elastic modulus, neither energy nor toughness showed early acceleration or saturation—highlighting their continuous enhancement as mineralization progresses. These findings underscore that while early tissue formation may quickly stabilize an implant mechanically, long-term resilience and durability depend on ongoing mineral deposition.

## Discussion

This study presents the first comprehensive biomechanical dissection of osseointegration, introducing a multidimensional framework that distinguishes between static outcomes and dynamic processes. It was evident that different evaluation metrics can yield highly distinct interpretations. Most notably, this study pioneers the application of speed metrics—a completely new dimension in implant research—providing insights not only into the extent of integration but also the rate and trajectory at which mechanical stability and biological attachment develop. These findings reveal that the timeline of osseointegration is not linear, and that both rate and quality of bone-implant integration are profoundly influenced by healing stage and surface characteristics.

A major conceptual advancement of this study lies in the contrast between traditional and functionally relevant mechanical parameters. Yield strength, the most commonly employed biomechanical metric in implant science, showed early saturation and limited correlation with the extent of mineralized tissue. In contrast, osseointegration energy—defined as the capacity of the implant-bone interface to absorb elastic deformation—exhibited continuous improvement throughout the healing period. This divergence introduces a critical paradigm shift. Energy, rather than yield strength, more accurately captures the functional integrity and durability of implant anchorage and should be recognized as a more clinically meaningful endpoint.

Furthermore, this study reveals for the first time the velocity and acceleration of osseointegration. By modeling biomechanical data through first- and second-degree equations, the speed of yield strength, modulus, energy, and toughness acquisition could be derived. UV photofunctionalization emerged as a transformative intervention—not merely accelerating early integration, but also elevating the final mechanical performance ceiling. The energy development rate was 4.9 times faster in UV-treated implants, with the energy value itself enhanced by 3.4-fold by week 3 compared to controls. These dynamic enhancements illustrate that UV photofunctionalization does not simply “speed up” healing—it creates a higher-performance interface capable of enduring greater mechanical demand over time.

The distinction between energy and toughness further deepens our understanding of biomechanical osseointegration. Osseointegration energy reflects the capacity of the implant interface to sustain elastic deformation, while toughness represents the total energy absorption until complete failure or rupture. Given that dental and orthopedic implants must operate within the elastic range under functional loading, energy is a more clinically and functionally relevant parameter than toughness. Importantly, a smaller gap between energy and toughness indicates a mature and resilient bone-implant interface—one that maximizes elastic performance while minimizing the risk of brittle failure. In both implant groups, toughness exceeded energy during the early to mid stages of healing, with energy progressively catching up over time. This convergence was more prominent in UV-photofunctionalized implants due to their exponential increase of energy, reflecting their accelerated and more complete osseointegration process.

The mechanical parameters used in this study demonstrated strong content, criterion, and construct validity. Content validity is evident in the comprehensive coverage of mechanical behaviors relevant to implant stability: strength, stiffness, energy absorption, and fracture resistance. Criterion validity was demonstrated by the clear and statistically significant differentiation between UV-treated and control implants across all metrics. Construct validity was confirmed by the logical and consistent relationships between mechanical performance and the degree of mineralized tissue, which were predicted by theory and aligned with the observed biological response. These findings validate the metrics not only as research tools but also as potential clinical benchmarks for future implant evaluation.

Another underappreciated finding is the evolution of elastic modulus over time. Elastic modulus—a marker of stiffness—was not static; it increased with healing time and was higher in UV photofunctionalized implants. This suggests that the quality of peri-implant bone, not just its quantity, is modulated by the surface properties of the implant. This aligns with prior studies showing that bone formed around rougher surfaces tends to be stiffer and harder due to its more advanced mineralization and denser collagen network [16, 18, 74]. Additionally, interfacial adhesion can be enhanced by surface roughness due to upregulated expression of adhesion proteins such as glycosaminoglycans/proteoglycans [17, 33]. These findings support the view that surface modifications, including UV treatment, may promote not only faster but functionally superior bone development and interfacial adhesion.

A critical insight from this study is the mismatch between yield strength and mineralized tissue area—an observation with profound implications for the field. Traditionally, bone-implant contact (BIC) percentage has been considered a central metric of osseointegration [75–80]. However, our data clearly show that yield strength exhibits a second-degree relationship with mineralized tissue, reaching a plateau beyond a certain threshold. This saturation suggests that BIC has a limited direct influence on mechanical strength beyond early stages. This finding uncovers a long-standing but previously unrecognized gap between morphological and mechanical integration. Importantly, osseointegration energy maintained a linear and proportional relationship with mineralized tissue area, underscoring its potential as a more informative and functionally relevant marker than BIC or yield strength. Thus, while BIC remains a useful morphological descriptor, this study supports a transition toward energy-based metrics for evaluating and predicting clinical performance.

Taken together, these findings advocate for a broader framework to evaluate implant success—one that integrates traditional biomechanical metrics with dynamic, time-resolved analyses. While peak values such as yield strength or removal torque remain important, they offer a limited snapshot of a complex, evolving biological process. The inclusion of energy and its rate of accumulation provides deeper insight into the functional resilience and adaptability of the bone–implant interface—parameters critical to the long-term performance of load-bearing implants. Clinically, this expanded perspective may inform early loading protocols, risk stratification in compromised bone conditions, and the design of next-generation implant surfaces. The regression models and numerical benchmarks established in this study offer a valuable reference point for future comparative evaluations across materials, geometries, and surface modifications.

At the same time, it is important to recognize the study’s limitations. The biomechanical assessment was based on static push-in testing, which does not fully replicate the dynamic mechanical environment of the oral cavity. Future studies incorporating cyclic or fatigue loading under physiologic conditions are essential to validate the predictive relevance of energy and speed metrics. Additionally, this study was conducted in a rat femur model using small-scale implants; translation to human clinical relevance will require validation in larger animal models, such as pigs or dogs, using full-size dental implants. Rather than replacing existing paradigms, the analytical framework proposed here is intended to augment conventional metrics—offering a more comprehensive and functionally meaningful approach to understanding and evaluating osseointegration.

## Conclusion

This in vivo biomechanical study redefines osseointegration as a dynamic, multidimensional process that extends beyond traditional strength-based assessments. By dissecting the distinct temporal trajectories of yield strength, elastic modulus, and energy absorption, the study introduces a novel analytical framework in which speed—the rate of biomechanical development—emerges as a critical, previously overlooked dimension of implant integration. This insight challenges the long-held belief that osseointegration stabilizes early and highlights a prolonged period of biomechanical advancement essential for long-term implant performance.

UV photofunctionalization is shown to be a transformative approach—not merely enhancing conventional biomechanical properties but fundamentally reshaping the trajectory and tempo of integration. It facilitates rapid early strength acquisition and drives a sustained, exponential increase in energy absorption, with the speed of energy gain elevated by up to 4.9-fold. Even elastic modulus, reflecting interfacial stiffness, demonstrates accelerated improvement, reinforcing the impact of surface physicochemical modification on both mechanical structure and function.

Importantly, this study reveals a dissociation between mineralized tissue area and strength parameters such as yield strength, while showing that energy correlates linearly with mineralization. This uncoupling exposes the limitations of strength-based metrics and underscores the need to adopt more functionally relevant indices. Taken together, these findings call for a paradigm shift in the evaluation of osseointegration—toward a framework that emphasizes mechanical durability, functional evolution, and clinical relevance.

## Data Availability

No datasets were generated or analysed during the current study.

## Abbreviations

BIC: bone-implant contact
Ca/P ratio: calcium-to-phosphorus ratio
Ca/Ti ratio: calcium-to-titanium ratio
EDX: energy-dispersive X-ray spectroscopy
SEM: scanning electron microscopy
UV: ultra violet
VUV: vacuum ultra violet
XPS: X-ray photoelectron spectroscopy
